# Hospice Use Among Medicare Beneficiaries With Parkinson Disease and Dementia With Lewy Bodies

**DOI:** 10.1001/jamanetworkopen.2025.0014

**Published:** 2025-03-04

**Authors:** Meredith Bock, Siqi Gan, Melissa Aldridge, Krista L. Harrison, Kristine Yaffe, Alexander K. Smith, John Boscardin, Lauren J. Hunt

**Affiliations:** 1 Division of Geriatrics, Department of Medicine, University of California, San Francisco; 2 Brookdale Department of Geriatrics and Palliative Medicine, Icahn School of Medicine at Mount Sinai, New York, New York; 3 Philip R. Lee Institute for Health Policy Studies, University of California, San Francisco; 4 Global Brain Health Institute, University of California, San Francisco; 5 Department of Neurology, University of California, San Francisco; 6 Department of Epidemiology and Biostatistics, University of California, San Francisco; 7 San Francisco Veterans Affairs Medical Center, San Francisco, California; 8 Department of Psychiatry, University of California, San Francisco; 9 School of Nursing, University of California, San Francisco

## Abstract

**Importance:**

Neurodegenerative disorders are now the most common reason that Medicare beneficiaries enroll in hospice for end-of-life care. People with all-cause dementia have high rates of suboptimal hospice use, but little is known about hospice use patterns in Lewy body disease, which includes both Parkinson disease (PD) and dementia with Lewy bodies (DLB).

**Objective:**

To compare patient characteristics, hospice agency characteristics, and patterns of use for beneficiaries with PD and DLB vs Alzheimer disease (AD).

**Design, Setting, and Participants:**

A retrospective cohort study including a 100% sample of national 2010-2020 calendar year Medicare data on hospice enrollees was performed. Data analysis was conducted from November 2023 to May 2024.

**Exposures:**

A diagnosis of PD or DLB, compared with AD as the reference group.

**Main Outcomes and Measures:**

Proportion of enrollees with short stays (<7 days), proportion with long stays (>180 days), proportion disenrolled for any reason before death, and disenrollment by type.

**Results:**

Of 11 327 324 Medicare beneficiaries enrolled in hospice between 2010 and 2020 who met eligibility criteria (mean [SD] age, 85.2 [7.5] years; 781 763 [63.0%] female), there were 958 182 (8.4%) with a primary diagnosis of AD, 232 864 (2.1%) with PD, and 49 340 (0.4%) with DLB. People with PD were more likely to experience a long stay (odds ratio [OR], 1.15; 95% CI, 1.13-1.16) compared with AD, whereas the odds for those with DLB were not increased. However, people with either PD or DLB were less likely to be disenrolled for extended prognosis compared with AD (OR for DLB, 0.82; 95% CI, 0.79-0.85; OR for PD, 0.86; 95% CI, 0.85-0.88). People with PD were more likely to revoke hospice (OR, 1.29; 95% CI, 1.27-1.32) compared with AD.

**Conclusions and Relevance:**

In this cohort study of Medicare beneficiaries, hospice use patterns differed by dementia subtype. Higher likelihood of hospice revocation in PD raises important questions about unmet needs and highlights the need for more research around the experience of the end of life in this growing population.

## Key Points

**Question:**

Does hospice use differ for Medicare beneficiaries with Lewy body disease (LBD), which includes both Parkinson disease (PD) and dementia with Lewy bodies (DLB), compared with Alzheimer disease (AD)?

**Findings:**

In this cohort study of 11 327 324 Medicare beneficiaries enrolled in hospice between 2010 and 2020, hospice enrollees with both PD and DLB were less likely to be disenrolled due to extended prognosis than those with AD. Enrollees with PD—but not DLB–were more likely to have longer lengths of stay and revoke hospice.

**Meaning:**

The findings of this study suggest a higher likelihood of revocation of hospice care in PD, raise important questions about their unmet needs in hospice, and highlight the need to disaggregate dementia subtypes for policy analysis.

## Teaser

This cohort study examines the use of hospice for individuals with Lewy body disease compared with those with Alzheimer disease.

## Introduction

Lewy body disease (LBD)—an umbrella term that includes Parkinson disease (PD) and dementia with Lewy bodies (DLB)—describes progressive, incurable neurodegenerative disorders.^1,2^ Parkinson disease is the second most common neurodegenerative disorder after Alzheimer disease (AD)^3,4,5^ and is the fastest growing neurologic disorder in the world.^1^ Parkinson disease and DLB exhibit common α-synuclein neuropathologic factors, but exist on a spectrum depending on whether motor or cognitive systems are affected first.^6^ End-stage LBD due to PD or DLB has a high burden of cognitive, psychiatric, mood, autonomic, and sensory symptoms.^7^ People with LBD experience decreased life span^8,9,10^ and a symptom burden equivalent to those with cancer.^11^ Palliative approaches in the outpatient setting are effective in improving patient quality of life, decreasing caregiver burden, and reducing bothersome symptoms in LBD.^12,13,14^ However, little is known about the experience of people with LBD enrolled in hospice for care at the end of life.

The Medicare Hospice Benefit was created for older adults with terminal illness (prognosis <6 months) who have opted out of disease-modifying treatments. This model is aligned with conditions such as cancer, where the decision to stop treatment typically leads to a brief and intensive end-of-life period. However, neurodegenerative illness is now the most common reason for hospice referral.^15^ Up to one-half of the people enrolled in hospice have a diagnosis of dementia^16^ and PD is the sixth most common reason for hospice referral.^15^ Neurodegenerative illnesses are slowly progressive over time with high levels of prognostic uncertainty,^17^ leading to increased rates of disenrollment (also referred to as live discharge) from hospice.^18^ Disenrollment decreases care continuity, imposes burdensome transitions of care, and decreases the likelihood that hospice will be involved in the last month of life when benefits are greatest.^19,20^

People with advanced PD and DLB represent a subpopulation of neurodegenerative diseases that pose even greater additional challenges for hospice care. They have a heterogeneous disease course with more psychiatric and behavioral symptoms,^21^ such as psychosis and attentional fluctuations, that providers of hospice care are less familiar with. In the few existing qualitative and small quantitative studies that explore the experience of people with LBD at the end of life,^22,23,24^ families describe a lack of support around advanced-care planning, confusion around the optimal timing of hospice, and inappropriate medication administration.^25,26^ However, to our knowledge, no study has described hospice use patterns for Medicare beneficiaries at a national level. Our aim was to assess patient characteristics, hospice agency characteristics, and patterns of use of people with LBD compared with AD in a national sample of Medicare beneficiaries. A better understanding of the current hospice use patterns and patterns in LBD may help identify unique needs and current gaps in care, and identity opportunities for improving end-of-life care for this growing but understudied population.

## Methods

### Data Sources and Participants

We used a 100% sample of national 2010-2020 Medicare data linked to databases of hospices and their regional characteristics (eFigure 1 in Supplement 1). We included Medicare beneficiaries admitted to hospice who died or were disenrolled between January 1, 2010, and December 31, 2020 (N = 12 423 832). We excluded individuals younger than 65 years at admission (n = 703 073) as this represents a different subpopulation eligible for Medicare due to specific illnesses and disabilities. We excluded beneficiaries from noncontiguous US states and territories because patterns of health care use and hospice availability are likely to be different. We restricted the sample to those with a primary hospice diagnosis of AD, PD, or LBD (n = 1 240 386). Analyses of reasons for disenrollment were restricted to the time period after September 1, 2012, as these data were only collected and reported for beneficiaries disenrolled after that date (n = 1 094 917). For each beneficiary, we identified the hospice providing care (via Centers for Medicare & Medicaid Services [CMS] provider numbers) and examined hospice characteristics (from the CMS Provider of Services files, the Hospice Public Use File, and the Hospice Compare database). We defined hospice region by linking hospice zip code to the Dartmouth Atlas. We obtained other regional data through the Neighborhood Atlas and the National Center for Health Statistics Urban/Rural Classification Scheme. This study was approved by the institutional review boards at the University of California, San Francisco, and the CMS Privacy Board. We received a waiver for consent as the data were collected as part of routine care and deidentified. Our study design and analysis adhere to all Strengthening the Reporting of Observational Studies in Epidemiology (STROBE) criteria for cohort studies.^27^

### Identification of Diagnosis

We identified diagnoses using *International Classification of Diseases, Ninth Revision* (*ICD-9*) and *International Statistical Classification of Diseases and Related Health Problems, Tenth Revision* (*ICD-10*) codes. For the PD cohort, we included beneficiaries with a primary hospice diagnosis of *ICD-9* code 332.0 (paralysis agitans) or *ICD-10* code G20 (PD), excluding beneficiaries with a diagnosis of secondary parkinsonism (332.1) or other degenerative diseases of the basal ganglia (*ICD-9* code 333.0). For the DLB cohort, we included individuals with a primary hospice diagnosis of DLB (*ICD-9* codes 331.82 or *ICD-10* code G31.38). For AD, we included individuals with a primary hospice diagnosis of AD (*ICD-9* code 331.0 or *ICD-10* codes G30.0, G30.1, G30.8, or G30.9). Those with multiple diagnoses were assigned to a group according to their primary hospice diagnosis.

### Outcomes

We defined suboptimal hospice use based on previously reported outcomes in the scientific and policy literature.^16,28,29^ These included (1) short stays (<7 days), as this leads to a large transition of care for families without enough time to fully benefit from hospice care; (2) long stays (>180 days), as this could reflect challenges with prognostication and increase the risk of disenrollment; and (3) disenrollment for any reason. We evaluated disenrollments within 7 days of admission and after 180 days, as well as the reason for disenrollment (revocation, extended prognosis or disqualification, movement out of service area, transfer to a different hospice, or discharged for cause). We ascertained the reason for disenrollment based on an algorithm using hospice claims (eTable 1 in Supplement 1).

### Covariates

Variables used to describe the sample included both patient and hospice agency characteristics. Decisions about which variables to include as covariates in our model were guided by theoretical approaches informed by previous research about hospice disenrollment.^30^ Patient-level characteristics were obtained from Medicare files, including race and ethnicity based on Research Triangle Institute race codes (American Indian or Alaska Native, Asian and Pacific Islander, Black, Hispanic, White, other, unknown),^27^ gender, Medicaid dual eligibility, Medicare Part C enrollment, comorbidities, care setting (home, nursing home, or assisted living facility) rural or urban location, Area Deprivation Index (calculated from zip codes), and region (East North Central, East South Central, Mid-Atlantic, Mountain, New England, Pacific, South Atlantic, West North Central, or West South Central). Hospice characteristics include hospice size, years in operation, and ownership type (for profit or nonprofit).

### Statistical Analysis

Data analysis was conducted from November 2023 to May 2024. We calculated descriptive statistics for beneficiaries and hospice agencies with PD, DLB, and AD. We used χ^2^ tests to evaluate differences between categorical variables and analysis of variance tests to evaluate differences for continuous variables between the groups, using AD as the reference group. The threshold for statistical significance was set at α = .05.

We plotted the total proportion of all hospice enrollees with AD, PD, and DLB as their primary diagnosis during the study period. We calculated this as the total number of individuals with AD, PD, or DLB who died or were disenrolled over the total number of all hospice enrollees with any diagnosis who died or were disenrolled annually. To evaluate whether primary diagnosis was associated with short stays, long stays, or disenrollment, we created multilevel logistic regression with random effects for hospice analyses to estimate the odds with primary diagnosis as the estimator variable (AD was set as the reference group) with random intercepts for hospice agencies. Model 1 was unadjusted, model 2 was adjusted for patient characteristics (age, gender, race and ethnicity, Medicaid dual eligibility, Medicare Part C enrollment, number of medical comorbidities, care setting, and urban or rural status). Model 3 was additionally adjusted for hospice characteristics (hospice size [quartile], years in operation, and ownership type). We generated estimated probabilities using the margins command with model 3. In a post hoc sensitivity analysis, we ran the model with hospice as a fixed effect and hospice as a random effect using linear regression. We conducted statistical analyses using SAS, version 9.4 M8 (SAS Institute) and Stata 18 (StataCorp LLC). Missingness for variables was 1% or less, so missing observations were excluded from the analysis.

We conducted 2 preplanned sensitivity analyses to evaluate the robustness of our logistic regression analysis. To address concerns around accuracy of the primary hospice diagnosis, we performed a sensitivity analysis limited to individuals who had both a diagnosis of AD, PD, or DLB as a primary hospice diagnosis and also had a diagnoses code for that condition in outpatient or inpatient Medicare claims in the 3 years preceding their hospice admission. To explore the regional differences in hospice billing for DLB as an allowable primary diagnosis due to Medicare Administrative Contractor oversight, we mapped the proportion of DLB as a primary hospice diagnosis by hospital referral region In a post hoc sensitivity analysis, we ran the fully adjusted model with hospice as a fixed effect and hospice as a random effect using linear regression and generated estimated probabilities from both of these models.

## Results

### Patient and Hospice Characteristics

Of the 11 327 324 Medicare beneficiaries enrolled in hospice between 2010 and 2020 (mean [SD] age, 85.2 [7.5] years; 781 763 [63.0%] female; 458 622 [37.0%] male), there were 958 182 (8.4%) with a primary diagnosis of AD, 232 864 (2.1%) with PD, and 49 340 (0.4%) with DLB (Table 1). Participants most commonly reported race as Black (6.9%), Hispanic (5.9%), and White (84.5%). Enrollees with PD or DLB were more likely to be enrolled in hospices that were in operation for a greater number of years and nonprofit.

**Table 1.** Baseline Characteristics of 1 240 386 Medicare Beneficiaries and Hospices by Dementia Subtype

Characteristic	No. (%)^a^			
	AD (n = 958 182)	PD (n = 232 864)	DLB (n = 49 340)	All (N = 1 240 386)
**Patients**				
Age, mean (SD), y	86.1 (7.3)	82.3 (7.1)	81.6 (7.4)	85.2 (7.5)
Race and ethnicity^b^				
American Indian or Alaska Native	2546 (0.3)	634 (0.3)	116 (0.2)	3296 (0.3)
Asian and Pacific Islander	16 846 (1.8)	4599 (2.0)	684 (1.4)	22 129 (1.8)
Black	73 631 (7.7)	9590 (4.1)	2220 (4.5)	85 441 (6.9)
Hispanic	57 586 (6.0)	13 342 (5.7)	1741 (3.5)	72 669 (5.9)
White	801 812 (83.7)	202 657 (87.0)	44 104 (89.4)	1 048 573 (84.5)
Other	4129 (0.4)	1382 (0.6)	263 (0.5)	5774 (0.5)
Unknown	1632 (0.2)	660 (0.3)	212 (0.4)	2504 (0.2)
Gender				
Female	664 676 (69.4)	94 870 (40.7)	22 217 (45.0)	781 763 (63.0)
Male	293 505 (30.6)	137 994 (59.3)	27 123 (55.0)	458 622 (37.0)
Medicaid dual eligible				
Yes	309 832 (32.3)	56 005 (24.1)	10 583 (21.4)	376 420 (30.4)
No	648 292 (67.7)	176 848 (75.9)	38 755 (78.5)	863 895 (69.7)
Enrolled in Medicare Part C				
Yes	298 974 (31.2)	70 183 (30.1)	15 406 (31.2)	384 563 (31.0)
No	659 150 (68.8)	162 670 (69.9)	33 932 (68.8)	855 752 (69.0)
No. of comorbidities				
0-2	391 793 (40.9)	92 664 (39.8)	22 297 (45.2)	506 754 (40.9)
3-4	350 440 (36.6)	85 941 (36.9)	17 643 (35.8)	454 024 (36.6)
≥5	215 891 (22.5)	54 248 (23.3)	9398 (19.0)	279 537 (22.5)
Residence type				
Home	350 766 (36.6)	105 777 (45.4)	20 391 (41.3)	476 934 (38.5)
Assisted living	197 068 (20.6)	32 052 (13.8)	9638 (19.5)	238 758 (19.3)
Nursing home	327 647 (34.2)	68 339 (29.3)	13 599 (27.6)	409 585 (33.0)
Other	82 030 (8.6)	26 569 (11.4)	5677 (11.5)	114 276 (9.2)
Rural/urban residence				
Rural	158 781 (16.6)	35 978 (15.5)	7812 (15.8)	202 571 (16.3)
Urban/suburban	798 191 (83.3)	196 559 (84.4)	41 476 (84.1)	1 036 226 (83.5)
Area Deprivation Index nation rank				
1-20 (Least deprived)	138 837 (14.5)	37 229 (16.0)	8014 (16.2)	184 080 (14.8)
21-40	224 544 (23.4)	57 336 (24.6)	12 947 (26.2)	294 827 (23.8)
41-60	266 905 (27.9)	66 164 (28.4)	14 784 (30.0)	347 853 (28.0)
61-80	253 710 (26.5)	57 999 (24.9)	11 076 (22.4)	322 785 (26.0)
81-100 (Most deprived)	62 424 (6.5)	11 751 (5.0)	2026 (4.1)	76 201 (6.1)
Region				
East North Central	158 905 (16.6)	38 883 (16.7)	9208 (18.7)	206 996 (16.7)
East South Central	66 981 (7.0)	13 628 (5.9)	2701 (5.5)	83 310 (6.7)
Mid-Atlantic	94 955 (9.9)	26 025 (11.2)	4351 (8.8)	125 331 (10.1)
Mountain	58 667 (6.1)	16 593 (7.1)	3293 (6.7)	78 553 (6.3)
New England	50 133 (5.2)	10 799 (4.6)	3045 (6.2)	63 977 (5.2)
Pacific	146 705 (15.3)	33 075 (14.2)	7840 (15.9)	187 620 (15.1)
South Atlantic	190 863 (19.9)	48 926 (21.0)	9767 (19.8)	249 556 (20.1)
West North Central	68 406 (7.1)	18 075 (7.8)	4656 (9.4)	91 137 (7.4)
West South Central	122 400 (12.8)	26 824 (11.5)	4477 (9.1)	153 701 (12.4)
Hospice years in operation				
0-5	131 333 (13.7)	27 454 (11.8)	5005 (10.1)	163 792 (13.2)
6-20	396 854 (41.4)	92 613 (39.8)	18 095 (36.7)	507 562 (40.9)
>20	428 717 (44.7)	112 564 (48.3)	26 205 (53.1)	567 486 (45.8)
Hospice size, No. of beneficiaries per year				
≤50	17 557 (1.8)	3156 (1.4)	515 (1.0)	21 228 (1.7)
51-500	369 162 (38.5)	80 868 (34.7)	16 494 (33.4)	466 524 (37.6)
>500	566 837 (59.2)	147 962 (63.5)	32 214 (65.3)	747 013 (60.2)
Hospice ownership				
Nonprofit	332 480 (34.7)	90 428 (38.8)	21 230 (43.0)	444 138 (35.8)
For profit	507 196 (52.9)	113 742 (48.8)	22 070 (44.7)	643 008 (51.8)
Government/other	117 228 (12.2)	28 461 (12.2)	6005 (12.2)	151 694 (12.2)

Abbreviations: AD, Alzheimer disease; DLB, dementia with Lewy bodies; PD, Parkinson disease.

^a^ All differences significant at *P* < .001.

^b^ Race and ethnicity categorization based on Research Triangle Institute race codes. There was no specification of ethnicities within the other category.^27^

### Hospice Use in Beneficiaries With AD, PD, and DLB

Table 2 summarizes hospice use patterns by dementia subtype. A lower proportion of people with LBD were disenrolled from hospice due to extended prognosis compared with AD (6.4% for PD, 5.8% for DLB, and 7.2% of AD). However, a higher proportion of people with PD revoked hospice care (7.0% for PD, 5.1% for DLB, and 5.4% for AD).

**Table 2.** Hospice Use Patterns for 1 240 386 Medicare Beneficiaries With PD, AD, and DLB

Characteristic	No. (%)			*P* value	Total, No. (%) (N = 1 240 386)
	AD (n = 958 182)	Lewy body condition			
		PD (n = 232 864)	DLB (n = 49 340)		
Median length of stay, d (IQR)	33 (8-139)	35 (9-140)	31 (9-122)	<.001	33 (9-138)
Mean length of stay, d (SD)	114.6 (190.4)	113.1 (183.5)	105.2 (117.0)	<.001	113.9 (188.7)
Proportion with short stays (<7 d)	190 416 (19.9)	43 339 (18.6)	9166 (18.6)	<.001	242 921 (19.6)
Proportion with long stays (>180 d)	187 442 (19.6)	45 685 (19.6)	8790 (17.8)	<.001	241 917 (19.5)
Proportion disenrolled					
Overall	182 173 (19.0)	45 023 (19.3)	8379 (17.0)	<.001	235 575 (19.0)
Disenrolled >180 d	68 712 (7.2)	14 924 (6.4)	2865 (5.8)	<.001	86 501 (7.0)
Disenrolled <7 d (early live discharges)	9225 (1.0)	2758 (1.2)	467 (1.0)	<.001	12 450 (1.0)
Disenrollment reason (for beneficiaries disenrolled after September 1, 2012)					
Revocation	45 966 (5.4)	13 876 (7.0)	2252 (5.1)	<.001	62 094 (5.7)
Extended prognosis/disqualification	79 295 (9.3)	15 470 (7.8)	3388 (7.7)	<.001	98 153 (9.0)
Movement out of service area	9461 (1.1)	2830 (1.4)	490 (1.1)	<.001	12 781 (1.2)
Transfer to different hospice	21 017 (2.5)	4873 (2.5)	1156 (2.6)	.09	27 046 (2.5)
Discharged for cause	2579 (0.3)	581 (0.3)	78 (0.2)	<.001	3238 (0.3)

Abbreviations: AD, Alzheimer disease; DLB, dementia with Lewy bodies; PD, Parkinson disease.

**Figure 1.**
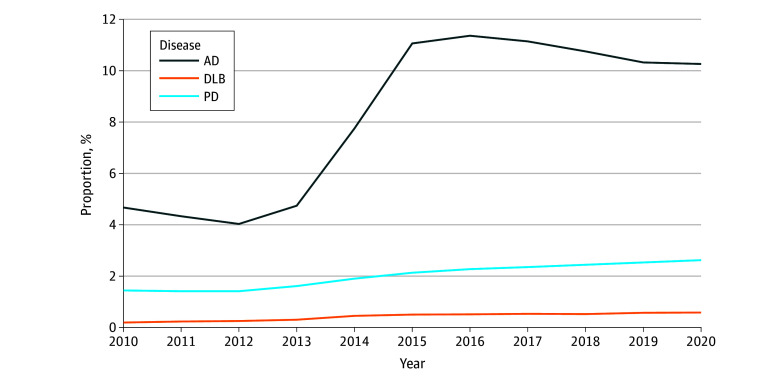
Proportion of All Hospice Enrollees With Alzheimer Disease (AD), Parkinson Disease (PD), or Dementia With Lewy Bodies (DLB) as a Primary Diagnosis, 2010-2020

The proportion of enrollments from hospice increased steeply for those with AD after 2013, whereas it increased at a slower rate for those with PD and LBD (Figure 1). Specifically, proportion of enrollments for AD increased from 4.7% in 2013 to 11.4% in 2016, whereas PD increased from 1.6% in 2013 to 2.3% and DLB increased from 0.3% in 2013 to 0.5% in 2016.

### Diagnosis and Types of Suboptimal Hospice Use

**Figure 2.**
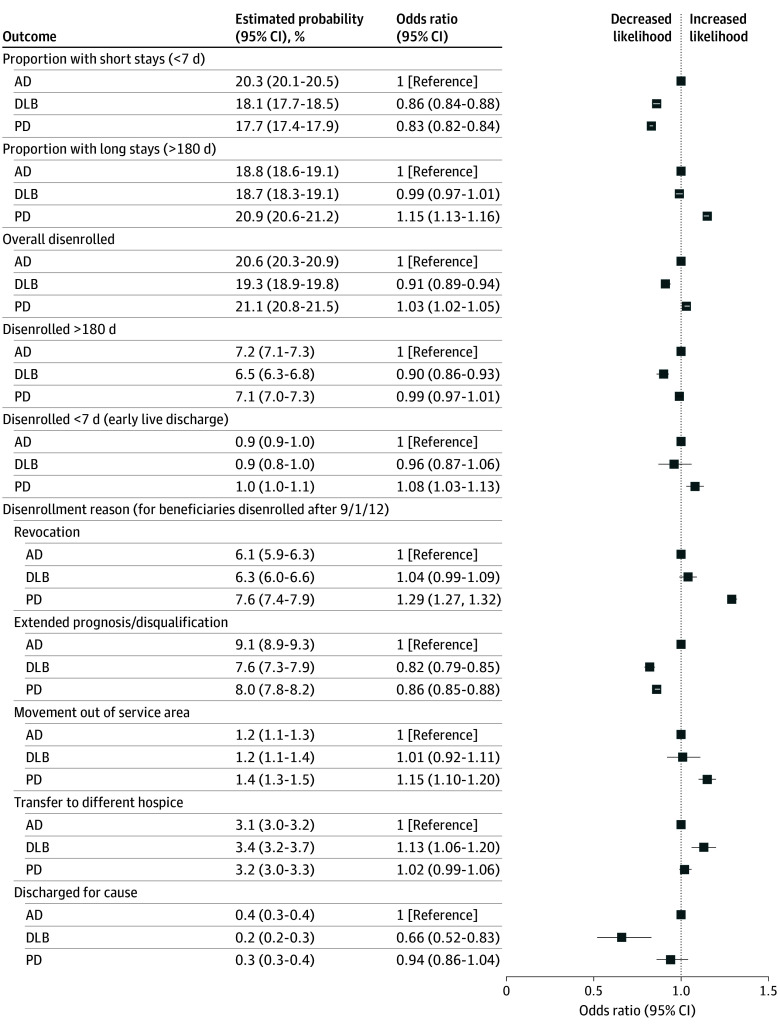
Association Between Dementia With Lewy Bodies (DLB) and Parkinson Disease (PD) Diagnosis Compared With PD Diagnosis With Suboptimal Hospice Use Alzheimer disease (AD) was used as the reference for all comparisons. Odds ratios represented are from the fully adjusted model in Table 3. Model 3 was adjusted for patient characteristics (age, gender, race and ethnicity, Medicaid dual eligibility, Medicare Part C enrollment, number of medical comorbidities, care setting, urban or rural status), and hospice characteristics (hospice age, hospice size, hospice type).

Table 3 and Figure 2 summarize the logistic regression analyses. In the fully adjusted model, people with DLB (odds ratio [OR], 0.86; 95% CI, 0.84-0.88; estimated probability, 18.1%) and PD (OR, 0.83; 95% CI, 0.82-0.84; estimated probability, 17.7%) had decreased likelihood of a short stay in hospice compared with people with AD. People with PD were more likely to experience a long stay (OR, 1.15; 95% CI, 1.13-1.16; estimated probability, 20.9%) compared with people with AD, whereas the odds for those with DLB were not increased. However, people with either PD or DLB were less likely to be disenrolled for extended prognosis compared with AD (OR for DLB, 0.82; 95% CI, 0.79-0.85; estimated probability, 7.6%; OR for PD, 0.86; 95% CI, 0.85-0.88; estimated probability, 8.0%). People with PD were more likely to revoke hospice (OR, 1.29; 95% CI, 1.27-1.32) or move out of their service area (OR, 1.15; 95% CI, 1.10-1.20; estimated probability, 1.4%) compared with people with AD.

**Table 3.** Association Between DLB and PD Diagnosis Compared With AD Diagnosis With Suboptimal Hospice Use

Variable	Model^a^		
	1	2	3
**Proportion with short stays (<7 d)**			
AD	1 [Reference]	1 [Reference]	1 [Reference]
DLB	0.89 (0.87-0.91)	0.86 (0.84-0.88)	0.86 (0.84-0.88)
PD	0.90 (0.89-0.91)	0.83 (0.82-0.85)	0.83 (0.82-0.84)
**Proportion with long stays (>180 d)**			
Overall			
AD	1 [Reference]	1 [Reference]	1 [Reference]
DLB	0.94 (0.91-0.96)	0.99 (0.97-1.02)	0.99 (0.97-1.01)
PD	1.02 (1.01-1.03)	1.14 (1.13-1.16)	1.15 (1.13-1.16)
**Proportion disenrolled**			
Overall			
AD	1 [Reference]	1 [Reference]	1 [Reference]
DLB	0.97 (0.95-0.99)	0.91 (0.89-0.93)	0.91 (0.89-0.94)
PD	1.06 (1.05-1.07)	1.03 (1.02-1.05)	1.03 (1.02-1.05)
Disenrolled >180 d			
AD	1 [Reference]	1 [Reference]	1 [Reference]
DLB	0.88 (0.85-0.92)	0.89 (0.86-0.93)	0.90 (0.86-0.93)
PD	0.91 (0.89-0.93)	0.99 (0.97-1.00)	0.99 (0.97-1.01)
Disenrolled <7 d (early live discharges)			
AD	1 [Reference]	1 [Reference]	1 [Reference]
DLB	1.06 (0.96-1.16)	0.94 (0.85-1.04)	0.96 (0.87-1.06)
PD	1.24 (1.19-1.30)	1.07 (1.02-1.12)	1.08 (1.03-1.13)
**Disenrollment reason (for beneficiaries disenrolled after September 1, 2012)**			
Revocation			
AD	1 [Reference]	1 [Reference]	1 [Reference]
DLB	1.14 (1.09-1.19)	1.03 (0.99-1.08)	1.04 (0.99-1.09)
PD	1.42 (1.39-1.45)	1.29 (1.26-1.32)	1.29 (1.27-1.32)
Extended prognosis/disqualification			
AD	1 [Reference]	1 [Reference]	1 [Reference]
DLB	0.82 (0.79-0.85)	0.82 (0.79-0.85)	0.82 (0.79-0.85)
PD	0.82 (0.81-0.84)	0.86 (0.84-0.88)	0.86 (0.85-0.88)
Movement out of service area			
AD	1 [Reference]	1 [Reference]	1 [Reference]
DLB	1.17 (1.06-1.28)	1.01 (0.92-1.11)	1.01 (0.92-1.11)
PD	1.32 (1.26-1.38)	1.15 (1.10-1.20)	1.15 (1.10-1.20)
Transfer to different hospice			
AD	1 [Reference]	1 [Reference]	1 [Reference]
DLB	1.21 (1.13-1.28)	1.11 (1.05-1.19)	1.13 (1.06-1.20)
PD	1.04 (1.01-1.08)	1.02 (0.98-1.05)	1.02 (0.99-1.06)
Discharged for cause			
AD	1 [Reference]	1 [Reference]	1 [Reference]
DLB	0.69 (0.55-0.87)	0.66 (0.52-0.82)	0.66 (0.52-0.83)
PD	0.98 (0.89-1.08)	0.95 (0.86-1.05)	0.94 (0.86-1.04)

Abbreviations: AD, Alzheimer disease; DLB, dementia with Lewy bodies; PD, Parkinson disease.

^a^ Model 1 is unadjusted. Model 2 is adjusted for patient characteristics (age, gender, race and ethnicity, Medicaid dual eligibility, Medicare Part C enrollment, number of medical comorbidities, care setting, and urban or rural status). Model 3 is additionally adjusted for hospice characteristics (hospice age, hospice size, and hospice type).

### Sensitivity Analyses

In a sensitivity analysis to ensure that hospice primary diagnoses match beneficiary prehospice diagnoses of dementia, we restricted our analyses to individuals with an *ICD* code indicating AD, PD, or DLB in the 3 years before hospice admission. Of 189 457 beneficiaries with PD admitted to hospice after 2013, 142 412 (75.2%) had PD *ICD* codes in inpatient, outpatient, and carrier file 3 years earlier. Of 42 468 beneficiaries with DLB admitted to hospice after 2013, 24 190 (57.0%) had LBD *ICD* codes indicating inpatient, outpatient, and carrier file 3 years earlier. Of 826 588 beneficiaries with AD admitted to hospice after 2013, 491 937 (59.5%) had AD *ICD* codes in inpatient, outpatient, carrier file 3 years prior. In this restricted sample, our model estimates were unchanged (eTable 2 in Supplement 1).

As there may be regional differences in hospice billing for DLB as an allowable primary diagnosis due to Medicare Administrator Contractor oversight, we mapped the proportion of DLB as a primary hospice diagnosis by hospital referral region. There were no clear geographic patterns (eFigure 2 in Supplement 1). Sensitivity analyses of different modeling approaches with hospice fixed or random effects resulted in similar estimated probabilities for outcomes (eTable 3 in Supplement 1).

## Discussion

In a 100% sample of Medicare hospice enrollees, we found distinct hospice use patterns for individuals with LBD compared with AD. Beneficiaries with PD and DLB had a lower risk of hospice disenrollment due to extended prognosis compared with AD, while PD was a risk factor for longer hospice stays. People with PD had 30% increased odds of revocation of hospice (OR, 1.29; 95% CI, 1.27-1.32), raising important questions about possible unmet needs. The proportion of individuals with PD and DLB using hospice increased steadily over the study period but did not dramatically shift after the 2013 CMS policy changes (excluding failure to thrive as an allowable hospice diagnosis) in the same way that AD did.^31^ Our study provides novel evidence that hospice use patterns differ by dementia subtype and should be disaggregated for policy analyses.

To our knowledge, this is the first nationally representative study exploring hospice use patterns in Medicare beneficiaries with LBD, a high-risk and understudied subpopulation of people with neurodegenerative illness. Our findings add to the current literature. Studies to date exploring hospice use in people with LBD have been qualitative,^22,23^ not nationally representative,^24^ or lacking in information about disenrollment.^32^ National studies of hospice use focus on all-cause dementia. This growing body of literature has shown that people with all-cause dementia encounter numerous barriers in the traditional hospice model, including uncertain prognosis, high need for caregiver support, lack of advanced care planning discussions before hospice admission, and heavy reliance on proxy decision-makers who may be unprepared for their roles.^33^ These challenges have led to high rates of disenrollment from hospice for people with dementia, which are particularly pronounced in those with more chronic conditions, younger age, female sex, lower income, and racial and ethnic minority status.^18^ However, to our knowledge, there have been no prior investigations into how hospice use patterns vary by diagnostic subtype.

Our finding that people with LBD have a lower risk of disenrollment due to extended prognosis is notable, especially because PD was a risk factor for longer hospice stay. People with DLB have shorter survival than AD (median, 3-4 years after diagnosis^24,34,35,36^), which likely accounts for the lower likelihood of disenrollment due to extended prognosis. People with LBD typically have more intensive symptom management needs, which may enable hospices to more easily justify continued enrollment. High prognostic variability, particularly for PD, may account for the longer stays seen in PD. The reasons for this are not understood, but could reflect the fact that individuals with better access to health care due to geographic region or socioeconomic status may be more likely to have LBD diagnosed and also have better access to high-quality hospice care.

The higher likelihood of revocation in PD raises the question of whether hospice is meeting important needs for this population at the end of life. Many of the medications in typical hospice comfort kits, such as neuroleptics^37,38,39^ and antiemetics, can acutely worsen parkinsonism or even lead to untimely death.^39^ People with PD are also at high risk of inappropriate deprescribing of dopaminergic medications, which may be seen as disease-directed but in fact are essential for pain and mood management, and acetylcholinesterase inhibitors, which can markedly help with psychiatric and behavioral symptoms. Families may notice problems with symptom management and revoke hospice care.

Our study additionally observed how changes in CMS regulation of hospice coding practices are associated with the prevalence of different diagnoses in the data. The 2013 CMS hospice policy change (to remove debility, failure to thrive, and certain dementia types from the list of eligible hospice diagnosis)^31^ and 2016 CMS hospice policy change (to reduce payments to hospice for stays >60 days)^39^ did not lead to as much code-shifting behavior for DLB and PD compared with AD. There is clear variation in coding practices for dementia, suggesting a need for further clarification so individuals with less common subtypes receive the appropriate attention in research studies.

Our analysis has several clinical implications. Given that PD is the most rapidly growing neurologic disorder, it is critical to increase education about LBD across the health care system, generate specific treatment protocols, and decrease ruptures in care continuity. Education and training programs increase clinical knowledge and confidence of those caring for people with dementia in hospice^40^; there is an unmet need for similar programs for LBD. Second, there is a need for research about how novel outpatient dementia care management programs, such as the Guiding an Improved Dementia Experience model for Medicare beneficiaries,^41^ can meet the palliative needs of individuals with dementia in the outpatient setting prior to hospice eligibility and lead to hospice referral timing that optimizes quality of care.

### Limitations

There are several limitations of our study. Lewy body disease is often underdiagnosed or misdiagnosed as AD,^42^ which may have biased our results toward the null. Our study is limited by diagnosis according to *ICD* codes. However, our sensitivity analyses also found associations when we compared the principal hospice diagnosis codes with the codes in the outpatient files 3 years preceding hospice enrollment. While we expect most individuals with PD advanced enough to be in hospice would have cognitive impairment based on prior research,^43^ the population of people with PD in this study may be cognitively heterogeneous.

## Conclusions

The findings of this cohort study suggest that hospice use patterns differ by dementia subtype and should be disaggregated for policy analyses. Higher rates of revocation for people with PD and DLB raise concerns about unmet needs in hospice. There is a need for more data around the hospice experience of people with LBD and their care partners, best practices, and education programs for providers of hospice care to address the needs of this quickly growing patient population.

## Notes

Supplement 1. **eFigure 1.** Derivation of the Analytic Cohort **eTable 1.** Algorithm to Identify Hospice Disenrollment **eTable 2.** Sensitivity Analysis for the Logistic Regression Analysis Restricted to Beneficiaries With PD, AD, or DLB ICD Codes in Outpatient, Inpatient, or Carrier File 3 Years Prior **eTable 3.** Sensitivity Analysis Converting the Model to a Linear Regression With Fixed Effects, Linear Regression With Mixed Effects, and Logistic Regression With Mixed Effects **eFigure 2.** Proportion of Hospice Enrollees With Dementia With Lewy Bodies by Hospital Referral Region

Supplement 2.
Data Sharing Statement
