# Supplementary material for: Hospice Use Among Medicare Beneficiaries With Parkinson Disease and Dementia With Lewy Bodies
Source: JAMA Netw Open. 2025 Mar 4;8(3):e250014. doi: 10.1001/jamanetworkopen.2025.0014 (PMC11880951; doi:10.1001/jamanetworkopen.2025.0014)
Supplement: Supplement 1. — eFigure 1. Derivation of the Analytic Cohort eTable 1. Algorithm to Identify Hospice Disenrollment eTable 2. Sensitivity Analysis for the Logistic Regression Analysis Restricted to Beneficiaries With PD, AD, or DLB ICD Codes in Outpatient, Inpatient, or Carrier File 3 Years Prior eTable 3. Sensitivity Analysis Converting the Model to a Linear Regression With Fixed Effects, Linear Regression With Mixed Effects, and Logistic Regression With Mixed Effects eFigure 2. Proportion of Hospice Enrollees With Dementia With Lewy Bodies by Hospital Referral Region [file jamanetwopen-e250014-s001.pdf]

Supplementary Online Content

Bock M, Gan S, Aldridge M, et al. Hospice use among Medicare beneficiaries with Parkinson disease and dementia with Lewy bodies. *JAMA Netw Open*. 2025;8(3):e250014. doi:10.1001/jamanetworkopen.2025.001

eFigure 1. Derivation of the Analytic Cohort

eTable 1. Algorithm to Identify Hospice Disenrollment

eTable 2. Sensitivity Analysis for the Logistic Regression Analysis Restricted to Beneficiaries With PD, AD, or DLB ICD Codes in Outpatient, Inpatient, or Carrier File 3 Years Prior

eTable 3. Sensitivity Analysis Converting the Model to a Linear Regression With Fixed Effects, Linear Regression With Mixed Effects, and Logistic Regression With Mixed Effects

eFigure 2. Proportion of Hospice Enrollees With Dementia With Lewy Bodies by Hospital Referral Region

This supplementary material has been provided by the authors to give readers additional information about their work.

© 2025 Bock M et al. *JAMA Network Open.*

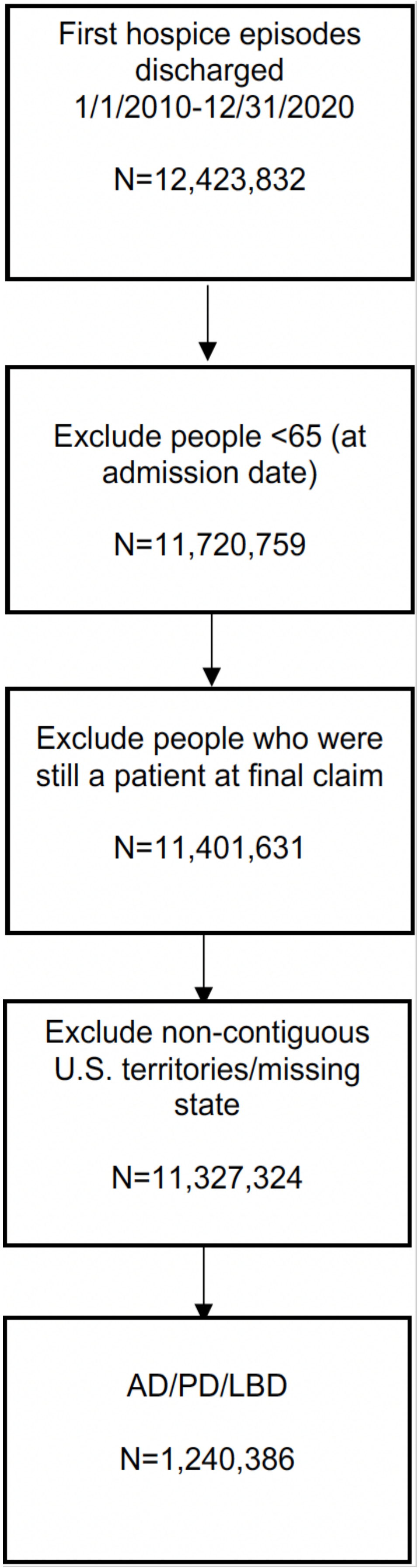

eFigure 1: Derivation of the analytic cohort.

© 2025 Bock M et al. *JAMA Network Open.*

eTable 1: Algorithm to identify hospice disenrollment

Outcome	Data Source	Variable Name	Operational Definition	Notes
Hospice Final Disposition at 1-Year Following Initial Admission	Hospice claims file (base claims file)	NCH_PTNT_STATUS_IND_CD	Died (NCH Patient Status Indicator Code=B)<br>Disenrolled (NCH Patient Status Indicator=A)<br>Still a Patient (NCH Patient Status Indicator=C)	
Disenrollment Reason				Based on Medicare Claims Processing Manual
Extended Prognosis	Hospice claims files (base claims file, condition code file, and occurrence code file)	N/A	NCH_PTNT_STATUS_IND_CD=A but does not have code for revocation or transferred/moved/disenrolled for cause in base claims, condition code, or occurrence file	
Revoked	Occurrence code file	CLM_RLT_OCRNC_CD	NCH_PTNT_STATUS_IND_CD=A and CLM_RLT_OCRNC_CD=42	
Transferred to another hospice	Base claims file	PTNT_DSCHRG_STUS_CD	NCH_PTNT_STATUS_IND_CD=A and PTNT_DSCHRG_STUS_CD=50 or 51	
Moved out of service area/not transferred to another hospice	Condition code file	CLM_RLT_COND_CD	NCH_PTNT_STATUS_IND_CD=A and CLM_RLT_COND_CD=52	
Discharged for cause	Condition code file	CLM_RLT_COND_CD	NCH_PTNT_STATUS_IND_CD=A and CLM_RLT_COND_CD=H2	

Note: For patients who fell into multiple categories of disenrollment based on occurrence codes (e.g. have both discharge code = 50-transfer, and condition code=52-move), the priority is revoke > move/discharge for cause > transfer

© 2025 Bock M et al. *JAMA Network Open.*

eTable 2: Sensitivity analysis for the logistic regression analysis restricted to beneficiaries with PD, AD, or DLB ICD codes in outpatient, inpatient, or carrier file 3 years prior.*

	Model 3	Model 3 (restricted diagnoses)
Proportion with short stays (<7 days)		
AD	ref.	ref.
DLB	0.86 (0.84, 0.88)	0.89 (0.86, 0.92)
PD	0.83 (0.82, 0.84)	0.86 (0.85, 0.88)
Proportion with long stays (>180 days)		
Overall		
AD	ref.	ref.
DLB	0.99 (0.97, 1.01)	0.96 (0.93, 1.00)
PD	1.15 (1.13, 1.16)	1.09 (1.07, 1.11)
Proportion Disenrolled		
Overall		
AD	ref.	ref.
DLB	0.91 (0.89, 0.94)	0.89 (0.86, 0.92)
PD	1.03 (1.02, 1.05)	1.02 (1.020, 1.04)
Disenrolled >180 days		
AD	ref.	ref.
DLB	0.90 (0.86, 0.93)	0.83 (0.78, 0.88)
PD	0.99 (0.97, 1.01)	0.95 (0.92, 0.97)
Disenrolled <7 days (early live discharges)		
AD	ref.	ref.
DLB	0.96 (0.87, 1.06)	0.94 (0.82, 1.08)
PD	1.08 (1.03, 1.13)	1.11 (1.05, 1.18)
Disenrollment reason (for beneficiaries disenrolled after 9/1/12)		
Revocation		
AD	ref.	ref.
DLB	1.04 (0.99, 1.09)	1.01 (0.95, 1.08)
PD	1.29 (1.27, 1.32)	1.30 (1.27, 1.34)
Extended prognosis/Disqualification		
AD	ref.	ref.
DLB	0.82 (0.79, 0.85)	0.81 (0.74, 0.85)
PD	0.86 (0.85, 0.88)	0.85 (0.83, 0.87)

© 2025 Bock M et al. *JAMA Network Open.*

Movement out of service area		
AD	ref.	ref.
DLB	1.01 (0.92, 1.11)	0.90 (0.79, 1.03)
PD	1.15 (1.10, 1.20)	1.10 (1.04, 1.17)
Transfer to different hospice		
AD	ref.	ref.
DLB	1.13 (1.06, 1.20)	1.09 (1.00, 1.19)
PD	1.02 (0.99, 1.06)	0.99 (0.95, 1.03)
Discharged for cause		
AD	ref.	ref.
DLB	0.66 (0.52, 0.83)	0.58 (0.42, 0.81)
PD	0.94 (0.86, 1.04)	0.92 (0.81, 1.05)

*Of 189457 beneficiaries with PD admitted to hospice after 2013, 142412 (75%) had PD ICD codes in inpatient, outpatient, carrier file 3 years prior. Of 42468 beneficiaries with DLB admitted to hospice after 2013, 24190 (57%) had LBD ICD codes in inpatient, outpatient, carrier file 3 years prior. Of 826588 beneficiaries with AD admitted to hospice after 2013, 491937 (60%) had AD ICD codes in inpatient, outpatient, carrier file 3 years prior. Model 3 adjusts for age, gender, race, Medicaid dual eligibility, Medicare Part C enrollment, number of medical comorbidities, care setting, urban/rural status, hospice age, hospice size, and hospice type.

© 2025 Bock M et al. *JAMA Network Open.*

eTable 3: Sensitivity analysis converting the model to a linear regression with fixed effects, linear regression with mixed effects, and logistic regression with mixed effects.

	linear regression-fixed effect** (patient+hospice covariates)	linear regression-mixed effect** (patient+hospice covariates)	logistic regression-mixed effect** (patient+hospice covariates)
Proportion with short stays (<7 days)			
AD	20.21% (20.13%, 20.29%)	20.29% (20.11%, 20.48%)	20.27% (20.07%, 20.48%)
LBD	17.80% (17.46%, 18.15%)	18.03% (17.65%, 18.42%)	18.09% (17.71%, 18.46%)
PD	17.39% (17.23%, 17.55%)	17.56% (17.32%, 17.79%)	17.66% (17.43%, 17.89%)
Proportion with long stays (>180 days)			
AD	19.10% (19.02%, 19.17%)	18.80% (18.53%, 19.06%)	18.83% (18.58%, 19.09%)
LBD	19.08% (18.73%, 19.43%)	18.64% (18.21%, 19.07%)	18.69% (18.27%, 19.11%)
PD	21.15% (20.98%, 21.31%)	20.77% (20.47%, 21.07%)	20.89% (20.58%, 21.21%)
Proportion Disenrolled			
Overall			
AD	18.79% (18.71%, 18.87%)	21.47% (21.07%, 21.86%)	20.63% (20.33%, 20.94%)
LBD	17.64% (17.30%, 17.98%)	20.18% (19.67%, 20.70%)	19.31% (18.86%, 19.76%)
PD	19.31% (19.15%, 19.47%)	21.91% (21.49%, 22.33%)	21.15% (20.80%, 21.49%)
Disenrolled >180 days			
AD	6.97% (6.91%, 7.02%)	7.41% (7.22%, 7.59%)	7.19% (7.05%, 7.33%)
LBD	6.39% (6.17%, 6.61%)	6.74% (6.45%, 7.03%)	6.51% (6.25%, 6.77%)
PD	6.92% (6.81%, 7.03%)	7.31% (7.10%, 7.52%)	7.13% (6.96%, 7.30%)
Disenrolled <7 days (early live discharges)			
AD	0.95% (0.93%, 0.97%)	0.96% (0.92%, 0.99%)	0.94% (0.91%, 0.97%)
LBD	0.94% (0.85%, 1.03%)	0.91% (0.82%, 1.00%)	0.91% (0.82%, 0.99%)

© 2025 Bock M et al. *JAMA Network Open.*

PD	1.05% (1.00%, 1.09%)	1.04% (0.99%, 1.09%)	1.02% (0.97%, 1.07%)
Disenrollment reason (for beneficiaries disenrolled after 9/1/12)*			
Revocation			
AD	5.36% (5.31%, 5.41%)	7.62% (7.29%, 7.94%)	6.13% (5.95%, 6.30%)
LBD	5.58% (5.37%, 5.79%)	7.78% (7.40%, 8.17%)	6.34% (6.03%, 6.64%)
PD	6.81% (6.70%, 6.91%)	9.03% (8.69%, 9.37%)	7.64% (7.40%, 7.88%)
Extended prognosis/Disqualification			
AD	9.18% (9.12%, 9.24%)	8.87% (8.66%, 9.08%)	9.07% (8.88%, 9.27%)
LBD	7.63% (7.37%, 7.90%)	7.33% (6.99%, 7.66%)	7.63% (7.34%, 7.92%)
PD	8.02% (7.89%, 8.15%)	7.71% (7.47%, 7.95%)	7.97% (7.77%, 8.18%)
Movement out of service area			
AD	1.13% (1.11%, 1.16%)	1.34% (1.25%, 1.43%)	1.22% (1.14%, 1.31%)
LBD	1.15% (1.05%, 1.25%)	1.33% (1.20%, 1.47%)	1.24% (1.10%, 1.37%)
PD	1.31% (1.26%, 1.36%)	1.50% (1.40%, 1.61%)	1.39% (1.28%, 1.49%)
Transfer to different hospice			
AD	2.44% (2.40%, 2.47%)	3.99% (3.82%, 4.17%)	3.09% (2.98%, 3.21%)
LBD	2.75% (2.60%, 2.89%)	4.28% (4.06%, 4.50%)	3.45% (3.22%, 3.67%)
PD	2.50% (2.43%, 2.57%)	4.05% (3.87%, 4.24%)	3.16% (3.02%, 3.30%)
Discharged for cause			
AD	0.30% (0.29%, 0.31%)	0.44% (0.37%, 0.51%)	0.35% (0.31%, 0.39%)
LBD	0.21% (0.16%, 0.26%)	0.35% (0.27%, 0.43%)	0.24% (0.18%, 0.29%)
PD	0.28% (0.26%, 0.31%)	0.42% (0.35%, 0.49%)	0.33% (0.29%, 0.38%)

**adjusted for age, gender, race, Medicaid dual eligibility, Medicare Part C enrollment, number of medical comorbidities, care setting, urban/rural status, hospice age, hospice size, hospice type

© 2025 Bock M et al. *JAMA Network Open.*

eFigure 2: Proportion of hospice enrollees with dementia with Lewy Bodies by hospital referral region.

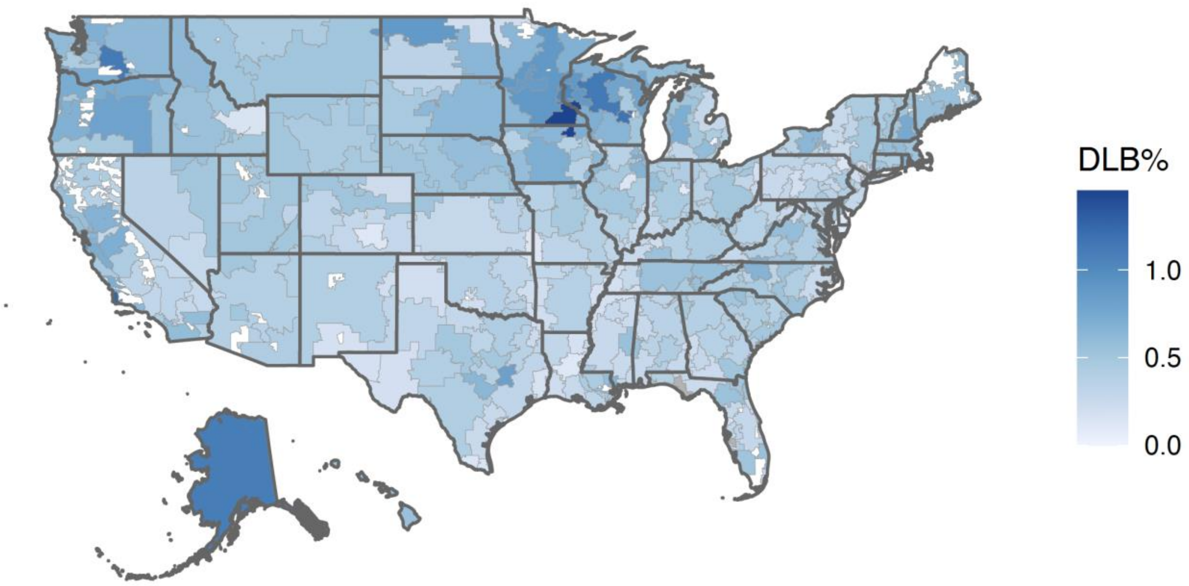

© 2025 Bock M et al. *JAMA Network Open.*
