# Supplementary material for: Hospice Use Among Medicare Beneficiaries With Parkinson Disease and Dementia With Lewy Bodies
Source: JAMA Netw Open. 2025 Mar 4;8(3):e250014. doi: 10.1001/jamanetworkopen.2025.0014 (PMC11880951; doi:10.1001/jamanetworkopen.2025.0014)
Supplement: Supplement 2. — Data Sharing Statement [file jamanetwopen-e250014-s002.pdf]

# Data Sharing Statement

Bock. Hospice Use Among Medicare Beneficiaries With Parkinson Disease and Dementia With Lewy Bodies. *JAMA Netw Open*. Published March 04, 2025.
doi:10.1001/jamanetworkopen.2025.0014

**Data**
**Data available:** No

**Additional Information**
**Explanation for why data not available:** Data access requires VRDC access per Medicare regulations.
